# How Is the Presence of Company Related to Thwarted Belongingness in Real Time? Taking a Closer Look at the Conceptualization of the Construct of the Interpersonal Theory of Suicide

**DOI:** 10.3390/ijerph17134873

**Published:** 2020-07-06

**Authors:** Nina Hallensleben, Heide Glaesmer, Thomas Forkmann, Dajana Rath, Maria Strauss, Anette Kersting, Lena Spangenberg

**Affiliations:** 1 Department of Medical Psychology and Medical Sociology, University of Leipzig, 04103 Leipzig, Germany; heide.glaesmer@medizin.uni-leipzig.de (H.G.); Lena.Spangenberg@medizin.uni-leipzig.de (L.S.); 2 Department of Clinical Psychology, University of Duisburg-Essen, 45141 Essen, Germany; thomas.forkmann@uni-due.de (T.F.); dajana.rath@uni-due.de (D.R.); 3 Department of Psychiatry, University of Leipzig, 04103 Leipzig, Germany; maria.strauss@medizin.uni-leipzig.de; 4 Department of Psychosomatic Medicine and Psychotherapy, University of Leipzig, 04103 Leipzig, Germany; anette.kersting@medizin.uni-leipzig.de

**Keywords:** interpersonal theory of suicide, thwarted belongingness, depression, company, partnership, intrapersonal factors, interpersonal factors, ecological momentary assessments, inpatients

## Abstract

(1) Background: The role of thwarted belongingness (TB) in predicting suicidal ideation, as originally assumed by the Interpersonal Theory of Suicide, is repeatedly challenged by empirical findings. This could be due to an inadequate conceptualization of the construct of TB that is assumed to be influenced by intrapersonal and interpersonal factors. (2) Methods: We examined the associations of TB with intrapersonal variables related to depression, and with interpersonal variables related to an individual’s actual social environment. We analyzed data from an ecological momentary assessment study in psychiatric inpatients with depressive disorders. *N* = 73 participants rated momentary TB, depressive affect and status of company up to 10 times per day, over a period of six days, on smartphones. (3) Results: TB was lower when assessed while participants were in company compared to when they were alone, and the more desired the company was, the less TB was experienced. Individuals who had a partnership experienced less momentary TB. Furthermore, higher levels of momentary depressive affect, as well as more stable levels of depression, were related to higher levels of TB, and the relation between the presence of company and TB was weaker for more depressed persons. (4) Conclusions: Our findings can be seen as evidence that both intrapersonal and interpersonal factors relate to TB, and thus support the conceptualization of TB as proposed by the Interpersonal Theory of Suicide.

## 1. Introduction

The Interpersonal Theory of Suicide (IPTS) [1] is one of the most popular recent frameworks for explaining the transition from suicidal ideation to suicidal behavior. It posits that individuals will engage in suicidal behavior when the desire for suicide coincides with the capability to act on their thoughts. In this regard, the IPTS provides the theoretical framework for distinguishing between individuals exclusively having suicidal thoughts and individuals conducting suicidal behavior. Further advantages of the IPTS include its clearly defined constructs, in conjunction with relatively straightforward assumptions regarding how these interact and relate to suicidality. Two interpersonal factors are assumed to cause suicidal desire: thwarted belongingness (TB) and perceived burdensomeness (PB). TB describes the unmet human need to belong that is expressed in feelings of social exclusion or social alienation. PB means the perception of being a burden to others, and the fatal conclusion that one’s own death would be a relief to others. According to the IPTS, suicidal desire is only translated into suicidal behavior if an individual possesses the capability to overcome the innate instinct for survival. This capability for suicide (CS) is meant to be acquired through repeated exposure to painful and provocative events (e.g., physical abuse, combat exposure, past suicidal behavior), but might also have a genetic component [2], and manifests in fearlessness about death and increased pain tolerance. 

To date, there has been a vast amount of research examining the assumptions of the IPTS in various countries and samples. The majority of findings support the IPTS, i.e., that TB, PB and their interactions relate to suicidal ideation, and that the three-way-interaction of TB, PB and CS is associated with the number of previous suicide attempts. However, effect sizes are mostly small to moderate, and a number of studies also found that relations between the theory’s constructs and suicide outcomes that were not in line with the IPTS [3]. It was repeatedly shown that suicidal ideation and behavior are more strongly related with PB than with TB. In their systematic review of the main assumptions of the IPTS, Ma, Batterham, Calear and Han [4] noted that 83% of the empirical tests of the association between PB and suicidal ideation were significant, but only 40% of the tests of the association between TB and suicidal ideation were significant. Many studies, including measurements of TB and PB, found significant associations with suicidal ideation only for PB. In most studies that found a significant association between TB and suicidal ideation, this accounted for a smaller amount of variance in suicidal ideation than PB. It should be noted that the vast majority of the studies conducted so far were cross-sectional, and based on retrospective reports of suicidal ideation and behavior [4]. However, current findings from prospective studies point in the same direction. A recent study that examined real-time data (via smartphone-based ecological momentary assessment), concerning the associations of the interpersonal variables PB and TB with suicidal ideation, found that PB significantly predicted the level of passive and active suicidal ideation a few hours later, but TB did not [5]. However, another study, which assessed suicidal ideation and the interpersonal variables of the IPTS daily over the period of a psychiatric inpatient stay for 491 patients, reported a predictive value for TB, insofar as the levels of both TB and PB at admission predicted the level of suicidal ideation at discharge, which holds for TB even when adding suicidal ideation at admission to the model [6]. 

Although there are mixed findings, PB appears overall to be a more robust predictor of suicidal ideation than TB. Besides the possibility that TB is not related to suicidal ideation in the way assumed by the IPTS, it might be that it was not sufficiently operationalized, or that the measures used to assess TB (in most studies, the TB subscale of the Interpersonal Needs Questionnaire (INQ)) [7] misrepresent the construct. In order to approach this question and to provide an alternative instrument for the assessment of TB, Ma, Batterham, Calear and Sunderland [8] developed the Thwarted Belongingness Scale (TBS), capturing TB with items independent from those of the INQ TB subscale. Comparative evaluation of the two scales supported the validity of the TB subscale of the INQ, and tests of the IPTS’s hypotheses suggested a less strong relation between TB and measures of suicidality when compared to PB, no matter what instrument was used to measure TB. These findings indicate that the seeming superiority of PB in predicting suicide risk is probably not due to the inadequate assessment of TB. Another explanation could be that an inappropriate conceptualization of the TB construct is to blame for its weak associations with suicidal ideation. TB is defined as a state that arises when the “need to belong”, a fundamental human psychological need that was described by Baumeister and Leary [9], is unmet. It is composed of two sub-factors: first, loneliness, which is conceptualized as an affect-laden cognition that one has too few social relations, and second, the absence of reciprocally-caring relationships, in which individuals both feel cared about and take care of others. Most importantly, TB is assumed to be a dynamic cognitive-affective state that is influenced by intrapersonal (e.g., activated interpersonal schemas, such as the tendency to interpret others’ behavior as affection or rejection, and current emotional states, such as a depressed mood) and interpersonal factors (an individuals’ actual interpersonal environment, e.g., the number of persons in one’s social network) [10]. However, little research has been conducted so far that aims at empirically testing these theoretical assumptions. This is remarkable, since Ribeiro and Joiner already requested in 2009 that future research should examine potential underlying facets of the constructs TB and PB [11]. 

Some studies, though, have investigated underlying factors of TB, mostly in order to better understand the links between known risk factors and suicidal behavior or depression. It has already been demonstrated that TB is positively associated with depression [12] and social anxiety [13], as well as fear of negative evaluation [14], negative interpersonal trust [15], insomnia [16,17,18], and perceived illness stigma in youth with inflammatory bowel disease [19]. Further, it could be shown that TB is inversely related to involvement in extracurricular activities in young adults [20], social support resulting from the disclosure of a suicide attempt from students with a history of suicide attempts [21], and the existence of a partnership in gay men [22]. Moreover, mixed findings are already available for newer technologies, illustrating that the influence of online interactions on TB is still unclear. While Moberg and Anestis [23] found that negative interactions on social networking sites predicted TB, Ringer and Anestis [12] only detected the independent associations of negative face-to-face interactions, but not of negative online interactions, with higher levels of TB, and Chalker and Comtois [24] did not find an association between TB and social connectedness via a mobile phone. Overall, these findings mainly suggest relations between TB and variables based on dysfunctional (interpersonal) cognitions (representing intrapersonal factors), while it remains rather unclear if there are also associations with more objective criteria, such as the mere presence of another person (representing interpersonal factors). To the best of our knowledge, the relation between the presence of company and TB has not yet been investigated.

One reason for the widespread lack of research in this area is likely the difficulty of adequately capturing dynamic constructs such as TB. This methodological challenge has become manageable, since smartphone-based ecological momentary assessments (EMA) have become available for collecting variables repeatedly in real time. EMA enables us to capture conditions and experiences at the exact moment they occur, minimizing any memory effects that often arise with traditional clinical assessments. In addition, contextual factors can be taken into account, as data collection takes place in the participants’ natural environment [25]. All these advantages make EMA the ideal method for capturing dynamic processes that may vary over time. Results from previous EMA studies on the IPTS suggest that TB is actually subject to temporal fluctuations. It could be shown that TB fluctuates, sometimes dramatically, even over the short time-periods of a few hours, as well as over several days, and up to several weeks in psychiatric inpatients and suicide attempters [5,26], pointing out the importance of assessing the construct repeatedly in short time-frames to avoid missing important fluctuations. The extent of the fluctuation of TB varied considerably inter-individually, and was comparable with the fluctuation of depressive affect [5]. Up to now, TB has only been investigated in EMA studies with regard to its association with, and potential predictive value for, suicidal thoughts [5,6,26]. 

In the current study, we would therefore like to focus more on investigating under which intra- and interpersonal conditions TB is more or less pronounced. If it were possible to identify those factors influencing TB more precisely, potential approaches for the mitigation of TB as a risk factor in the development of suicidal thoughts could be derived. Analyzing data from an EMA study, we therefore examined whether intrapersonal variables related to depression, and interpersonal variables related to an individual’s actual social environment, affect self-reported TB in psychiatric inpatients. We have chosen variables that should represent the factors of interest (intra- and interpersonal ones as they have been proposed by the IPTS) as best as possible, and cover a wide range of relevant experiences and behaviors (e.g., we assume that depression is related to many intrapersonal influences on TB as formulated in the IPTS, such as the tendency towards negative evaluation), as well as being practicable to capture. The aims of the present study were to determine whether momentary self-reported TB is associated with (1) the momentary presence of another person, (2) the desirability of momentary company, (3) momentarily depressive affect, and (4) more stable (“trait-like”) intra- and interpersonal variables, namely depression and relationship status. 

Since there are only partial preliminary findings on these specific issues, we partly base our hypotheses on results from previous research, and partly, as best as possible, on the assumptions made in the IPTS. Accordingly, we assume that TB is negatively associated with the presence of another person. Further, we assume a negative relation between the desirability of company and TB. This is, as we expect to observe the presence of desired company being a positively evaluated interpersonal environment, and vice versa (the presence of undesired company as a negatively evaluated interpersonal environment). IPTS’s statements suggest that positive social interactions potentially reduce TB, while negative social interactions tended to be associated with the higher TB and loneliness measured in previous research [12,14,15,19,23]. In line with previous findings, we assume that depressive affect and trait-like depression is positively related to TB [12,13], and the existence of partnership is inversely related to TB [22].

## 2. Materials and Methods 

### 2.1. Participants and Procedure

A total of 74 patients from three German psychiatric hospitals took part in an EMA study for six consecutive days. Inclusion criteria were a primary diagnosis of unipolar depression (i.e., major depression or dysthymia), current or life-time suicidal ideation, age ≥ 18, and fluent German. Exclusion criteria were the diagnosis of bipolar affective disorder, psychotic symptoms, substance addiction, or an IQ < 85 according to a language based intelligence test [27]. We had to exclude one participant from the analyses, who had a missing value in one of the demographic variables of interest (relationship status). Therefore, the final sample consisted of *n* = 73. 

Of the included participants, *n* = 68 had a primary diagnosis of major depression, and *n* = 5 of dysthymia. Age was between 18 and 85 years (M = 37.6, SD = 14.4), the majority of the sample (*n* = 52, 71.2%) was female, 24 (32.9%) had a life partner and *n* = 26 (35.6%) reported one or more prior suicide attempts. Participants received up to EUR 50 for participation and completion of the study. All study procedures were approved by the Ethical Review Board of the Medical Faculty of University of Leipzig (No: 388-13-16122013). For a more detailed description of the study procedure, including ethical considerations, please refer to Forkmann et al. [28]. 

### 2.2. Assessments 

#### 2.2.1. Baseline Assessment

Participants underwent a structured clinical interview (SCID-I) [29] to ensure diagnosis of depression. Furthermore, they completed a number of questionnaires that assessed various clinical parameters, including the Rasch-based depression screening (DESC-II) [30,31] for the assessment of level of baseline depression, the Interpersonal Needs Questionnaire (INQ) [7,32] for the assessment of the level of baseline TB, and demographic characteristics such as relationship status. 

The sample exhibited a mean DESC-II value of 25.7 (SD = 6.5, range from 12 to 39), which is far above the proposed cut-off of 12 [31] for a depressive episode. The mean TB score of the sample was 4.1 (SD = 1.2, range from 2 to 8), which is above the 88th percentile, and thus above average compared to a representative non-clinical sample [33].

#### 2.2.2. EMA Assessment

EMA data collection was realized by using the software movisensXS [34] and Android smartphones that were lent to the participants for the duration of study assessment. Participants were signaled 10 times per day. Signals occurred randomly between 8:00 a.m. and 7:50 p.m. with a minimum interval of 30 minutes between measurement points. At every measurement point, participants were asked to rate their momentary level of passive and active suicidal ideation, depressive affect, hopelessness, PB, TB, and context factors such as the current status of company. 

For the present study, only ratings of current TB, depressive affect and company were analyzed. Two items assessed momentary levels of TB (“At this moment I feel lonely” and “At this moment I feel like I do not belong”), which were rated on a 5-point Likert scale (0—not at all to 4—extremely), resulting in a sum score (ranging from 0 to 8) with higher values indicating higher levels of TB. The items were based on the German TB scale of the INQ and adapted to the requirements of EMA (short and momentary wording). The two items that assessed depressive affect (“At this moment I feel downhearted” and “At this moment I feel sad”) were taken from PANAS-X [35] and were likewise adapted to EMA requirements in terms of the momentary wording. Analogous to the TB items, they were rated on a 5-point Likert scale (0—not at all to 4—extremely), resulting in a sum score (ranging from 0 to 8) with higher values indicating higher levels of depressive affect. A psychometric evaluation of the larger EMA item set utilized in the main study proved the good to excellent reliability values (on person level and on measurement level) as well as the convergent validity of the EMA items that assessed TB and depressive affect [28]. 

Regarding company, there were three items of interest: one dichotomous item to assess the presence of company at the time of the measurement point (“Was any other person with you at the time of the signal?”), and two items for the desirability status of the company (“if alone: I would rather be in company”; ”if in company: I would rather be alone”), which were rated on a 5-point Likert scale (0—not at all to 4—extremely). The last two items were combined into a total discrepancy value ranging from 0 (no discrepancy between real and desired status of company at all) to 4 (strong discrepancy between real and desired status of company).

### 2.3. Data Analysis

The dataset had a nested structure, with theoretical 60 (measurement points on level 1) × 73 (persons on level 2) = 4380 observations. The data analytic strategy followed two steps. For descriptive purposes, mean scores were first aggregated across all participants and measurement points, in order to examine the influence of the different conditions of company on TB in general. Second, we conducted multilevel analyses with TB sum scores as the outcome variable in order to test our hypotheses, taking into account the nested structure of the data. One multilevel model was computed that tested the associations between the presence of company, the desirability of company, depressive affect and TB (model 1). This model only contained predictors at the measurement level (level 1), and these were all person-mean centered, as model 1 examined within-person effects [36]. A second model was set up (model 2) that additionally included predictors at person level (level 2), namely the existence of partnership and baseline depression, while the level 1 predictors included in the model remained the same as in model 1. In model 2, the level 1 predictors were grand-mean centered to control for their effects, as we were particularly interested in between-person effects this time, and the continuous level 2 predictor (baseline depression) was also grand-mean centered to facilitate the interpretation of the parameter estimates, while the dichotomous 0/1-coded level 2 predictor (existence of partnership) was left in its raw metric [36]. All multilevel models were estimated by restricted maximum likelihood estimation since the number of level 2 units was relatively small [37]. For all models where deviance tests revealed better fits for the random slope model, when compared to the more restrictive random intercept model, random slopes were allowed. We used the statistical software HLM 7 [38] for conducting the multilevel analyses.

## 3. Results

### 3.1. Descriptive Statistics of the EMA Variables

Of the maximum possible 4380 measurement points, 3953 were valid. Thus, the average compliance with EMA assessments was about 90%, with a minimum of 75% and a maximum of 100%, as has already been reported [28]. 

Across all measurement points, participants indicated a mean TB sum score of 3.1 (SD = 2.6, range from 0 to 8). The intra-class correlation (ICC) for TB sum score was .57, indicating that 43% of its variance is accounted for by within-person variability. The mean score for depressive affect across all measurement points was 3.9 (SD = 2.5, range from 0 to 8).

**Figure 1:**
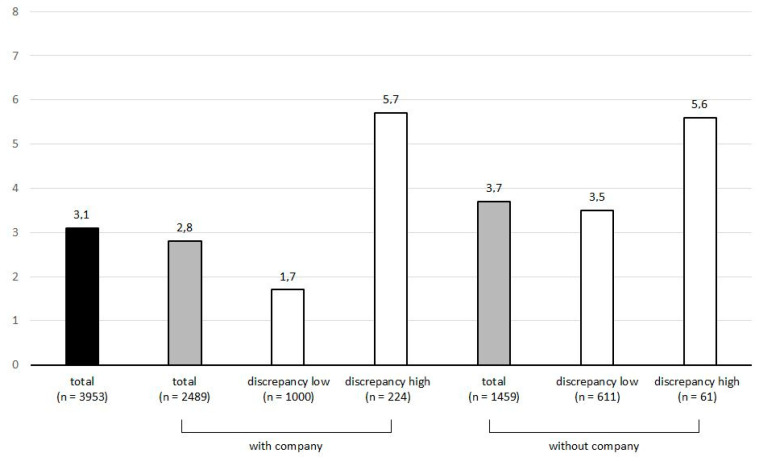
Mean values of thwarted belongingness for different conditions of company. Grey bars represent the variable ‘Presence of company’, and white bars represent the variable ‘Desirability of company’. Subsequent multilevel analysis tested whether the described differences were statistically significant.

Company was present at 63.0% (*n* = 2491) of the measurement points. The mean of the total discrepancy value, which indicates perceived discrepancy between the real and desired status of company (extracted from the items “I would rather be in company” if participants were alone at the time of measurement and “I would rather be alone” if participants were in company at the time of measurement) was 1.2 (SD = 1.3, range from 0 to 4). At 40.8% of all measurement points (*n* = 1611), there was no discrepancy at all between the real and desired status of company (discrepancy value of 0). At 23.0% of all measurement points (*n* = 909), there was a discrepancy value of 1. At 20.7% of all the measurement points (*n* = 818), there was a discrepancy value of 2. At 8.3% of all measurement points (*n* = 326), there was a discrepancy value of 3, and at only 7.3% (*n* = 287) there was a discrepancy value of 4, which was the maximum discrepancy between the real and desired status of company. The means and distributions of the discrepancy values were comparable for the two conditions of company (if alone and if in company). For all ‘currently alone’ measurement points, the mean discrepancy was 1.1 (SD = 1.2), and for all ‘currently in company’ measurement points the mean discrepancy was 1.2 (SD = 1.3). At 41.8% (n = 611) of those ‘currently alone’ measurement points, participants would not rather have been in company at all (discrepancy value of 0), and at only 4.2% (*n* = 62) of them did they have the strong wish to be in company instead (discrepancy value of 4). At 40.1% (*n* = 1000) of the ‘currently in company’ measurement points, participants would not rather have been alone at all, and at 9.0% (*n* = 225) of them, they had the strong wish to be alone instead. Interestingly, the mean TB values were nearly equal for those experiencing the strongest discrepancy (discrepancy value of 4) in both conditions, whereas the TB mean scores differed for the lowest discrepancy values (see Figure 1). 

Figure 1 illustrates the mean TB values for different conditions of company. It is intended to provide an overview of the associations between the company conditions and TB. In order to test these relations, the following multilevel models were set up. 

### 3.2. Multilevel Analyses

#### 3.2.1. Predictors at Measurement Level (Level 1)

The results of multilevel model 1 are shown in Table 1. All included level 1 predictors were significantly related with TB. There was a negative association with the presence of company, and positive associations with the discrepancy of company and depressive affect. 

#### 3.2.2. Additional Predictors at Person Level (Level 2)

Table 2 presents the results of multilevel model 2, where predictors at person level were added. The level 2 predictors were also significantly related to TB, while the relations at level 1 remained. There was a negative effect for the existence of partnership, and a positive effect for baseline depression.

#### 3.2.3. Testing for Cross-Level Interactions

As can be seen from the random effects in Table 1 and Table 2, the slopes for all predictors at level 1 significantly differed between the participants, i.e., the relations between presence of company and TB, between desirability of company and TB, and between depressive affect and TB were of varying strengths for the different persons. It would be interesting to examine which characteristics of the participants (i.e., variables at person-level) determined the strengths of these relations. For doing so, we conducted further multilevel analyses that focused on cross-level interactions between the concerned relations at level 1, and both predictors at level 2 that were included in our analyses. These additional models only comprised one predictor at level 1 and one predictor at level 2, which was added to the slope between the level 1 predictor and TB as only the moderating effects of the level 2 variables were of interest. All level 1 predictors were person-mean centered, since we were interested in cross-level effects, and the continuous level 2 predictor (baseline depression) was grand-mean centered, while the dichotomous level 2 predictor (existence of partnership) was left in its raw metric. This time we did not consider all predictors in one model, in order to avoid too much complexity in the models, and because it is difficult to interpret several cross-level interaction effects in one model [39]. Hence, we set up six additional models with TB as the outcome variable: 

(A.1) Presence of company as level 1 predictor, and existence of partnership as level 2 moderator in the relation of presence of company with TB,

(A.2) Presence of company as level 1 predictor, and baseline depression as level 2 moderator in the relation of presence of company with TB, 

(A.3) Desirability of company as level 1 predictor, and existence of partnership as level 2 moderator in the relation of desirability of company with TB,

(A.4) Desirability of company as level 1 predictor, and baseline depression as level 2 moderator in the relation of desirability of company with TB,

(A.5) Depressive affect as level 1 predictor, and existence of partnership as level 2 moderator in the relation of depressive affect with TB, and

(A.6) Depressive affect as level 1 predictor, and baseline depression as level 2 moderator in the relation of depressive affect with TB.

There was only one significant negative cross-level interaction effect, namely, baseline depression moderating the relation between presence of company and TB. The results of all additional multilevel analyses (models A.1–A.6) are displayed in Table A1, Table A2, Table A3, Table A4, Table A5 and Table A6 in Appendix A. 

## 4. Discussion

This study aimed at examining whether perceived TB, captured in real time via EMA, is affected by company or depressive affect. We were particularly interested in the associations of TB with current presence of another person and with the desirability of the current company. By investigating this issue, we are hoping to gain new insights into the conceptualization of the TB component of the IPTS, in order to finally obtain a better understanding of the construct. This may be necessary because the role of TB in predicting suicidal ideation, as originally assumed by the theory, has been repeatedly challenged by empirical studies [4]. Whether this is due to the lack of validity of TB or to the inadequate conceptualization of the construct is not yet clear. As yet, 10 years after Joiner published the IPTS, surprisingly little knowledge has been revised concerning the interpersonal constructs TB and PB. Therefore, to the best of our knowledge, this study is the first to address the question of whether perceived TB is influenced by actual company. 

Our results clearly show that company is related to reported TB, which applies to both the mere presence of company and the desirability of the company. 

### 4.1. Presence of Company

We found a negative association between the presence of company and TB. Apparently, as might be intuitively expected and as we have hypothesized, people generally experienced less TB when another person was present. This finding is particularly relevant for the distinction between TB and loneliness. A large body of studies has illustrated that loneliness is only partially influenced by objective social indicators, e.g., social network size [40,41,42,43,44]. Loneliness is therefore meant to reflect perceived social isolation, and is considered as highly subjective [40,45]. The fact that we found an effect of the presence of company, as an objective indicator of social connectedness, on TB could mean that the experience of TB actually exceeds feelings of loneliness, suggesting that the construct truly consists of several components, as described in the IPTS [10]: one intrapersonal component, that is influenced by subjective factors (e.g., cognitive evaluation of social contacts) and to a certain extent depicts loneliness, and one interpersonal component, that is influenced by objective factors (e.g., number of social connections) and to a certain extent depicts the social reality of an individual. In this respect, our results support the assumptions made in the IPTS about the underlying structure of TB. 

### 4.2. Desirability of Company

Furthermore, we found a positive association of discrepancy between the real and desired status of company and TB. This means the less desirable the currently present company was, the more TB was experienced, which is in line with our hypotheses. We expected that the presence of undesired company represents a negatively evaluated social interaction that should be associated with higher TB, according to previous research. While this is only an assumption in our case, the studies we referred to have mostly investigated situations with explicitly negative references, e.g., negative face-to-face [12] and online interactions [23], and explicitly measured negative states like fear of negative evaluation [14], negative interpersonal trust [15] and illness stigma [19]. Our results extend this previous evidence with the finding that the mere discrepancy between real and desired company, whether or not this is explicitly negatively evaluated, is associated with higher levels of TB. When interpreting these results, we should consider the possibility that the desirability of company, in the way it has been assessed in this study, could also be influenced by other factors, that are related to, for example, current mood, rather than to the current social encounter. Interestingly, our descriptive analyses showed that a high discrepancy between real and desired company was associated with approximately the same level of TB, regardless of whether one was in company or alone. Contrarily, in those cases where no discrepancy between real and desired company was reported, less TB was experienced when in company compared to when alone. These descriptive differences, as well as our multilevel results, indicate that the presence of company is not unconditionally related to TB, but that the relation depends on the desirability of company. This again points to the interplay of intra- and interpersonal factors associated with TB.

### 4.3. Depressive Affect

We found a positive relation between depressive affect and TB, indicating that the more depressed people reported to be at a certain moment, the more TB they coincidentally experienced. This is in line with previous research that found a relation between depression and TB, although this was previously only shown on retrospectively captured cross-sectional data [12,13]. We were able to confirm this preliminary finding, and to show that the relation also applies to momentarily captured real-time data.

### 4.4. Depression and Partnership at Person Level

All associations discussed so far have been related to the measurement level. Furthermore, we tested if more trait-like variables (at the person level) had an influence on momentarily rated TB. In fact, the level of baseline depression was positively associated, and being in a partnership was negatively associated with moment-by-moment TB. In other words, the more depressed a person has been during the last two weeks (time period covered by DESC-II that was used to assess baseline depression), the more momentary TB they reported, and persons that had a partnership at the time of baseline assessment experienced less momentary TB. These findings are consistent with previous research, that found a relation between depression and TB [12,13] and relationship status and TB [22]. Beyond that, we found that the level of baseline depression moderates the above-mentioned association of presence of company with TB, i.e., the more depressed a person had been during the previous two weeks, the weaker the relationship between current company and coincident TB. Overall, these findings illustrate that both momentary as well as temporally more stable variables relate to momentary TB. Furthermore, our findings suggest that both intrapersonal (depressive affect and baseline depression level) and interpersonal factors (presence of company and relationship status) are associated with the level of TB, as assumed by the authors of the IPTS [10], and that they even interact with each other. 

However, our analyses have shown that all relations found at the measurement level differed between the participants, which indicates that there might be other factors at the person level, which we have not considered, moderating these relations. These considerations illustrate the complexity of the mechanisms underlying the construct TB, and provide directions for further research. 

### 4.5. Strengths, Limitations and Directions for Further Research

The use of EMA is a major strength of the work presented here, as it enabled us to investigate the relation of the dynamic variable TB with its contextual factors repeatedly and in real time, making a reliable assessment of the construct possible. As far as we know, this is the first study investigating the associations between company and TB, and between depressive affect and TB, in real time.

Beside these strengths, our findings should be considered in the context of several limitations. First, by examining psychiatric inpatients currently suffering from a depressive disorder, we had a homogeneous but very specific sample, showing above-average values in TB. This aspect limits the generalizability of our findings, so further studies should examine more heterogeneous samples. 

Questionnaire data was used in order to include more stable (“trait-like”) variables in our analyses. However, it is doubtful to what extent those really capture traits, or at least fairly stable characteristics, since studies indicate that even constructs that are normally assessed via questionnaires (that sometimes cover long periods of time) can be subject to considerable and short-term fluctuations [5,46]. 

Since the focus of this study was on the analysis of inpatients’ EMA data, we mainly investigated temporary influences on TB. Our results are, in a manner of speaking, close-ups of current encounters, but they do not take into account an individual’s entire interpersonal background. Insofar, we could only test a few aspects of the conceptualization of TB. Overall, the selection of variables examined in this study is far from being exhaustive in order to investigate the issue of interest. This work can therefore only be understood as a first approach to the subject. Future studies should additionally have a greater focus on surveying the entire social network of a person by, for example, assessing the number of friends and acquaintances of the participants. 

## 5. Conclusions

The current EMA study examined whether the presence and the desirability of company, and depressive affect, were related to reported TB in psychiatric inpatients. In line with our hypotheses, we found the presence and higher desirability of company to be associated with lower levels of TB, and higher levels of depressive affect to be associated with higher levels of TB. Furthermore, we also found that more trait-like variables at the person level were related to moment-to-moment TB. In fact, we identified a negative association between the existence of partnership and TB, and a positive association between baseline depression and TB. Additionally, baseline depression was found to moderate the relation between the presence of company and TB, in the direction that the more depressive a person, the less strong the relation. 

Our findings can be seen as evidence that both interpersonal and intrapersonal factors are related to TB, as assumed by the authors of the IPTS [10]. Although the present work can only be understood as a first approach to the issue, it points to a proper conceptualization of the construct TB. In conjunction with findings supporting the validity of the instruments used to measure TB [8], our results indicate that TB may have only limited predictive value for suicidal ideation, and that PB should be prioritized as an interpersonal risk factor for suicidality. However, currently the evidence on the conceptualization of TB is too limited to draw firm conclusions.

If TB should indeed be a risk factor for suicidal ideation, as originally assumed by the IPTS [10], our findings could be used to derive interventions. We have found that the already-proven fluctuation of TB is related to both intra- and interpersonal factors in inpatients with depression. From a clinical perspective, this means that a change in these accompanying factors could lead to a change in TB, which could have beneficial effects on suicidal ideation, e.g., by focusing on depressive affect in therapy, or by recommending contact with others in critical situations as part of an emergency plan. Further research in this area is needed to continue with this promising approach.

## Figures and Tables

**Table 1:** Parameter estimates for multilevel model 1.

Predictors	Fixed Effects				Random Effects			
	Est.	SE	t(df)	*p*	Est.	SD	Χ^2^ (df)	*p*
Intercept	3.11	0.23	13.69 (72)	<0.001	3.78	1.94	8338.74 (71)	<0.001
Company	−0.48	0.08	−6.25 (72)	<0.001	0.27	0.52	190.03 (719)	<0.001
Discrepancy	0.26	0.03	9.53 (72)	<0.001	0.02	0.14	122.71 (71)	<0.001
Depressive affect	0.45	0.03	15.02 (72)	<0.001	0.05	0.23	377.38 (71)	<0.001

Notes: Outcome variable is TB. *n* = 3953. Est. = Estimate (unstandardized regression coefficient). SE = standard error.

**Table 2:** Parameter estimates for multilevel model 2.

Predictors	Fixed Effects				Random Effects			
	Est.	SE	t (df)	*p*	Est.	SD	Χ^2^ (df)	*p*
Intercept	3.46	0.2	17.27 (70)	<0.001	1.96	1.4	1882.88 (69)	<0.001
Partner	−0.88	0.33	−2.65 (70)	0.01				
Baseline depression	0.07	0.03	2.85 (70)	0.006				
Company	−0.48	0.08	−6.17 (70)	<0.001	0.27	0.52	189.67 (71)	<0.001
Discrepancy	0.26	0.03	9.48 (70)	<0.001	0.02	0.14	122.58 (71)	<0.001
Depressive affect	0.46	0.03	15.62 (70)	<0.001	0.05	0.22	375.60 (71)	<0.001

Notes: Outcome variable is TB. Predictors at level 2 are underlined. *n* (level 1) = 3953. *n* (level 2) = 73. Est. = Estimate (unstandardized regression coefficient). SE = standard error.

## Appendix A

**Table A1:** Parameter estimates for multilevel model A.1.

Predictors	Fixed Effects				Random Effects			
	Est.	SE	t (df)	*p*	Est.	SD	Χ^2^ (df)	*p*
Intercept	3.11	0.23	13.69 (72)	<0.001	3.77	1.94	5466.79 (72)	<0.001
Company	−0.58	0.1	−5.85 (71)	<0.001	0.35	0.59	173.10 (71)	<0.001
Partner	−0.02	0.21	−0.09 (71)	0.928				

Notes: Outcome variable is TB. *n* (level 1) = 3953. *n* (level 2) = 73. Est. = Estimate (unstandardized regression coefficient). SE = standard error.

**Table A2:** Parameter estimates for multilevel model A.2.

Predictors	Fixed Effects				Random Effects			
	Est.	SE	t (df)	*p*	Est.	SD	Χ^2^ (df)	*p*
Intercept	3.11	0.23	13.69 (72)	<0.001	3.77	1.94	5466.47 (72)	<0.001
Company	−0.58	0.09	−6.76 (71)	<0.001	0.31	0.56	162.81 (71)	<0.001
Baseline depression	−0.03	0.01	−2.23 (71)	0.029				

*Notes:* Outcome variable is TB. *n* (level 1) = 3953. *n* (level 2) = 73. Est. = Estimate (unstandardized regression coefficient). SE = standard error.

**Table A3:** Parameter estimates for multilevel model A.3.

Predictors	Fixed Effects				Random Effects			
	Est.	SE	t (df)	*p*	Est.	SD	Χ^2^ (df)	*p*
Intercept	3.11	0.23	13.69 (72)	<0.001	3.77	1.94	5816.76 (72)	<0.001
Discrepancy	0.37	0.05	7.14 (71)	<0.001	0.31	0.56	261.34 (71)	<0.001
Partner	0.05	0.09	0.59 (71)	0.558				

Notes: Outcome variable is TB. *n* (level 1) = 3953. *n* (level 2) = 73. Est. = Estimate (unstandardized regression coefficient). SE = standard error.

**Table A4:** Parameter estimates for multilevel model A.4.

Predictors	Fixed Effects				Random Effects			
	Est.	SE	t (df)	*p*	Est.	SD	Χ^2^ (df)	*p*
Intercept	3.11	0.23	13.69 (72)	<0.001	3.77	1.94	5816.11 (72)	<0.001
Discrepancy	0.38	0.04	9.14 (71)	<0.001	0.09	0.3	255.10 (71)	<0.001
Baseline depression	−0.00	0.01	−0.58 (71)	0.632				

Notes: Outcome variable is TB. *n* (level 1) = 3953. *n* (level 2) = 73. Est. = Estimate (unstandardized regression coefficient). SE = standard error.

**Table A5:** Parameter estimates for multilevel model A.5.

Predictors	Fixed Effects				Random Effects			
	Est.	SE	t (df)	*p*	Est.	SD	Χ^2^ (df)	*p*
Intercept	3.11	0.23	13.70 (72)	<0.001	3.78	1.94	8023.95 (72)	<0.001
Depressive affect	0.48	0.04	12.09 (71)	<0.001	0.06	0.25	466.09 (71)	<0.001
Partner	0.05	0.07	0.75 (71)	0.457				

*Notes:* Outcome variable is TB. *n* (level 1) = 3953. *n* (level 2) = 73. Est. = Estimate (unstandardized regression coefficient). SE = standard error.

**Table A6:** Parameter estimates for multilevel model A.6.

Predictors	Fixed Effects				Random Effects			
	Est.	SE	t (df)	*p*	Est.	SD	Χ^2^ (df)	*p*
Intercept	3.11	0.23	13.70 (72)	<0.001	3.78	1.94	8023.62 (72)	<0.001
Depressive affect	0.5	0.03	15.44 (71)	<0.001	0.06	0.25	460.82 (71)	<0.001
Baseline depression	−0.00	0.01	−0.24 (71)	0.809				

*Notes:* Outcome variable is TB. *n* (level 1) = 3953. *n* (level 2) = 73. Est. = Estimate (unstandardized regression coefficient). SE = standard error.
